# APOL1 High-Risk Genotype is Not Associated With New or Worsening of Proteinuria or Kidney Function Decline Following COVID-19 Vaccination

**DOI:** 10.1016/j.ekir.2024.06.023

**Published:** 2024-06-20

**Authors:** Sarah E. Nystrom, Karen L. Soldano, Micki Rockett, Somenath Datta, Guojie Li, Daniel Silas, Melanie E. Garrett, Allison E. Ashley-Koch, Opeyemi A. Olabisi

**Affiliations:** 1 Division of Nephrology, Duke Molecular Physiology Institute, Duke University School of Medicine, Durham, North Carolina, USA; 2 Duke Clinical and Translational Science Institute, Duke University School of Medicine, Durham, North Carolina, USA

**Keywords:** African American, APOL1, COVID-19, proteinuria, SARS-CoV-2, vaccine

## Abstract

**Introduction:**

SARS-CoV-2 infection increases systemic inflammatory cytokines which act as a second-hit driver of Apolipoprotein L1 (APOL1)-mediated collapsing glomerulopathy. SARS-CoV-2 vaccination also increases cytokines. Recent reports of new glomerular disease in individuals with *APOL1* high-risk genotype (HRG) following SARS-CoV-2 vaccination raised the concern SARS-CoV-2 vaccination may also act as a second-hit driver of APOL1-mediated glomerulopathy.

**Methods:**

We screened 1507 adults in the Duke’s Measurement to Understand Reclassification of Disease of Cabarrus and Kannapolis (MURDOCK) registry and enrolled 105 eligible participants with available SARS-CoV-2 vaccination data, prevaccination and postvaccination serum creatinine, and urine protein measurements. Paired data were stratified by number of APOL1 risk alleles (RAs) and compared within groups using Wilcoxon signed rank test and across groups by analysis of variance.

**Results:**

Among 105 participants, 30 (28.6%) had 2, 39 (37.1%) had 1, and 36 (34.3%) had 0 APOL1 RA. Most of the participants (94%) received at least 2 doses of vaccine. Most (98%) received the BNT162B2 (Pfizer) or mRNA-1273 (Moderna) vaccine. On average, the prevaccine and postvaccine laboratory samples were drawn 648 days apart. There were no detectable differences between pre- and post-serum creatinine or pre- and post-urine albumin creatinine ratio irrespective of the participants’ APOL1 genotype. Finally, most participants with APOL1 RA had the most common haplotype (E150, I228, and K255) and lacked the recently described protective N264K haplotype.

**Conclusion:**

In this observational study, *APOL1* HRG is not associated with new or worsening of proteinuria or decline in kidney function following SARS-CoV-2 vaccination. Validation of this result in larger cohorts would further support the renal safety of SARS-CoV-2 vaccine in individuals with APOL1 HRG.

## Graphical abstract

**Figure:**
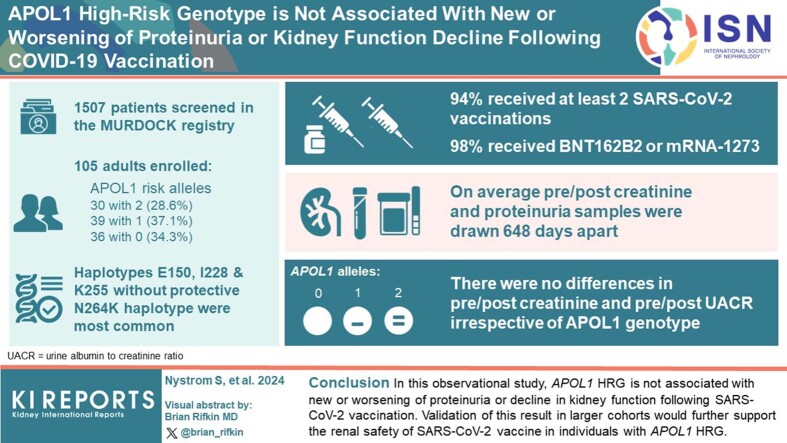

Concern about the potential adverse effects of the SARS-CoV-2 vaccine was a major contributor to its initial low uptake and skepticism about its safety persists among patients today.^1^ The vaccine’s proven efficacy in reducing severity of disease and death from COVID-19 have not silenced these concerns. An estimated 30% of eligible Americans remain unvaccinated or have not been fully vaccinated against SARS-CoV-2.^1^, ^2^, ^3^ Recent case reports of new onset glomerular disease in temporal association with SARS-CoV-2 vaccination brought renewed concerns about the vaccine’s safety in predisposed populations with established immune-mediated glomerular disease or a genetic susceptibility.^4^, ^5^, ^6^, ^7^, ^8^, ^9^, ^10^, ^11^, ^12^, ^13^, ^14^, ^15^ Individuals with *APOL1* HRG (G1G1, G1G2, and G2G2) are one such population. When SARS-CoV-2 infects individuals with *APOL1* HRG, their risk for developing collapsing glomerulopathy (also called COVID-19-associated nephropathy, or COVAN) is increased.^16^^,^^17^ Moreover, *APOL1* HRG is present in more than 90% of COVAN cases despite being found in only 12% to 15% of the African American population.^17^^,^^18^ Our recent investigation of the link between COVID-19, *APOL1* HRG, and COVAN shows that COVID-19-induced cytokines synergistically drive APOL1 expression and cytotoxicity in human kidney cell models.^19^ Consistent with this result, we also found that COVAN is associated with high expression of APOL1 protein in the podocytes and glomerular endothelial cells of kidney biopsies. Together with previous knowledge that iatrogenic interferons cause collapsing glomerulopathy in patients with *APOL1* HRG, these recent findings strongly suggest that cytokines induced by COVID-19 act as second-hit drivers of COVAN.^20^^,^^21^

SARS-CoV-2 vaccines, like SARS-CoV-2 infection, also cause an increase in systemic cytokines. However, the cytokine spike induced by the vaccine is transient, unlike the sustained cytokine activation that can be associated with SARS-CoV-2 infection.^22^, ^23^, ^24^ A recent study found that increases in IFN-gamma, IL-15, and CXCL10 comprise the defining cytokine signature following SARS-CoV-2 vaccination, with IFN-gamma levels increased up to 20-fold over baseline after the second dose of vaccine.^22^ Considering that IFN-gamma is the most potent known inducer of APOL1 expression in human podocyte models,^19^^,^^20^ these findings provoke the question, do SARS*-*CoV*-2* vaccines increase the risk of new-onset podocytopathy in people with *APOL1* HRG? The answer to this question is important for the estimated 6.2 million African Americans and tens of millions of people around the world with *APOL1* HRG.^25^

To date, there are no studies about the impact of *APOL1* HRG on the risk of SARS-CoV-2 vaccine-associated glomerular disease. Recent case reports of new or relapsing glomerular disease temporally associated with SARS-CoV-2 vaccination include focal segmental glomerulosclerosis, minimal change disease, IgA nephropathy, antineutrophilic cytoplasmic antibody-associated glomerulonephritis, membranous nephropathy, lupus nephritis, and anti-glomerular basement membrane disease.^4^, ^5^, ^6^^,^^14^^,^^15^ African American/Black persons with *APOL1* HRG are underrepresented in these studies. Notably, a series of 29 cases of biopsy-proven glomerular disease in patients recently vaccinated against SARS-CoV-2 identified 2 occurrences of collapsing glomerulopathy in African American/Black individuals with *APOL1* HRG.^6^ Therefore, it remains unknown whether SARS-CoV-2 vaccination increases risk for kidney disease among patients who carry *APOL1* HRG.

To investigate this question, we measured serum creatinine and urine protein before and after SARS-CoV-2 vaccination between October 2020 and March 2023 in a cohort of African American/Black adults in North Carolina. We report our findings that *APOL1* HRG is not associated with new kidney disease in this patient cohort.

## Methods

### Study Design, Participants, and Electronic Medical Record Review

We performed an observational review of data from participants from 2 cohorts (DARB and MURDOCK) enrolled through collaboration with MURDOCK registry, a 12,526-participant community-based registry covering a 20-zip code region in North Carolina. Both cohorts drew from the registry subgroup of 1507 participants who self-identify as African American/Black without overlap in cohort participation. Written informed consent for genotyping and genetic analysis was obtained from all participants and the study was approved by the Duke University Institutional Review Board.

Initial analysis focused on eligible participants within the Duke APOL1 Research Biorepository, NCT04160507 (DARB cohort), a previously identified cohort of self-identified African American/Black participants, aged 50 years and older, with known *APOL1* genotype, no history of kidney disease or diabetes, normal baseline serum creatinine, and urine protein-to-creatinine ratio (UPCR) of less than 300 mg/g. To increase robustness of sample size and provide inclusion of additional *APOL1* genotypes, study eligibility criteria were expanded to enroll additional African American/Black adults, aged 18 years and older, with baseline eGFR greater than 15, no history of kidney transplant, and for whom *APOL1* genotype was previously unknown (MURDOCK cohort).

Participants meeting eligibility criteria for DARB were consented to and enrolled between October 2020 and December 2021. All DARB participants provided or had biobanked urine and blood samples for measurement of baseline UPCR and serum creatinine. *APOL1* genotyping was performed at the time of DARB enrollment. Participants with 2 *APOL1* RAs were recontacted between May 2022 and August 2022 to obtain interval history of SARS-CoV-2 vaccination (including vaccine manufacturer, number of vaccines received, and exact dates of vaccine administration) as well as blood and urine samples for repeat measurement of serum creatinine and UPCR.

Participants meeting eligibility criteria for the MURDOCK cohort were consented to at the time of MURDOCK registry enrollment between February 2009 and February 2016. Electronic health record (EHR) review was performed on the 1185 qualifying individuals in April 2023. Individuals meeting MURDOCK cohort eligibility criteria who had available documentation of SARS-CoV-2 vaccination (vaccine manufacturer, number of vaccines received, and exact dates of vaccine administration), available pre/post vaccination laboratory values (serum creatinine +/− urine protein data), available biobanked whole blood samples, and consent for genetic sequencing were included in final study.

All remaining DARB cohort participants, regardless of *APOL1* genotype, who were identified to have both documented COVID-19 vaccination data (vaccine manufacturer, number of vaccines received, and exact dates of vaccine administration) and pre/post-vaccination laboratory values (serum creatinine +/− urine protein data) searchable in the EHR in MURDOCK’s database were also included for final study.

### DNA Extraction and *APOL1* Genotyping

Genomic DNA was extracted from peripheral blood mononuclear cells or from whole blood using NucleoSpin Tissue kit (Macherey-Nagel, cat no. 740952) per manufacturer’s protocol. *APOL1* genotyping was performed using Applied Biosystems Taqman allelic discrimination assays for G1 single nucleotide polymorphism (p.Ser342Gly) and G2 six bp deletion (p.Asn388_Tyr389del) using QuantStudio 6 Flex System. This assay has 100% analytic specificity and an analytic sensitivity (limit of detection) of 1.0ng DNA for the detection of *APOL1* risk variants in DNA extracted from peripheral blood mononuclear cells. *APOL1* genotype result from Taqman allelic discrimination assays were confirmed by direct sequencing. *APOL1* haplotype E150K, M228I, R255K, and N264K were determined by next-generation sequencing (Azenta Life Sciences).^26^^,^^27^

### Measurement of UPCR and Serum Creatinine

DARB participant UPCR and serum creatinine measurements were performed by Labcorp Corporation in North Carolina.

### Conversion Equation

For the 16 DARB participants with paired measures of UPCR, albuminuria levels were estimated using proteinuria via a previously published conversion equation by Sumida *et al.*^28^^,^^29^

### Analysis

Descriptive characteristics were compared using Fisher exact test for categorical variables, and either analysis of variance or the nonparametric Kruskal-Wallis test for continuous variables depending on normality (GraphPad Prism 10.2.0 software). Paired prevaccine and postvaccine serum creatinine and urine protein quantification data were stratified by number of RAs and the nonparametric Wilcoxon signed rank test was performed for each risk group to assess the difference between post versus prevaccine measures. Change scores were additionally calculated for each individual’s paired laboratory measurements (prevaccine measurement subtracted from postvaccine measurement) and graphed in box plot or violin format to show the distribution around 0 (median, lower quartile, upper quartile, minimum, and maximum). Change scores were stratified by the number of RAs and compared across 3 groups (2 RAs, 1 RA, and 0 RAs) using analysis of variance. All graphs and statistics were performed using GraphPad Prism 10.2.0 software. Significance was set at *P* < 0.05 for all tests.

## Results

### Participants, *APOL1* Genotype, and Vaccine Characteristic

Of the 1507 MURDOCK registrants who self-identified as African American/Black, 175 participants were eligible for DARB. Twenty-six of these 175 were identified to carry 2 *APOL1* RAs after genotyping and were recontacted for inclusion. Twenty-one provided vaccination history (vaccine manufacturer, number of vaccines received, and exact dates of vaccine administration) and completed postvaccine laboratory testing. Twelve of the other 149 eligible participants had both documented COVID-19 vaccination data and pre/post-vaccination laboratory values (serum creatinine and/or urine protein data) searchable via EHR review in MURDOCK’s database.

**Figure 1:**
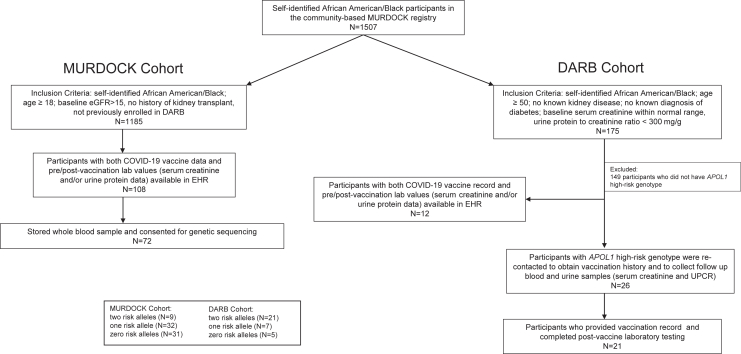
Enrollment flow chart. APOL1, Apolipoprotein L1; DARB, Duke APOL1 Research Biorepository; eGFR, estimated glomerular filtration rate; EHR, electronic health record; MURDOCK, Measurement to Understand Reclassification of Disease of Cabarrus and Kannapolis; UPCR, urine protein-to-creatinine ratio.

Subsequent EHR review identified an additional 1185 registrants meeting criteria from the MURDOCK cohort, after exclusion of all DARB participants. Of these 1185 individuals, 108 participants had both documentation of COVID-19 vaccination data and pre/post-vaccination laboratory values (serum creatinine and/or urine protein data) searchable via EHR. Among these 108, 72 participants were identified with available biobanked whole-blood samples and consent for genetic sequencing. These 72 individuals were genotyped for APOL1 and included for final analysis. The enrollment flow chart is presented in Figure 1.

A total of 105 patients were included in the final study, with 33 enrolled from the DARB cohort and 72 enrolled from the MURDOCK cohort (Figure 1). In the pooled population, 30 patients were identified to have 2 *APOL1* RAs, 39 patients with 1 *APOL1* RA, and 36 patients with 0 *APOL1* RA. The median age across all study participants and within each RA stratification was 62 years. In the group with 2 *APOL1* RAs, mean age was 63 years (range, 42–81) with 7 male participants (23%). By comparison, mean age for the group with 1 *APOL1* RA was 61 years (range, 35–85) with 11 male participants (28%), and mean age for the group with 0 *APOL1* RA was 63 years (range, 32–83) with 11 male participants (31%).

All participants self-identified as African American/Black. *APOL1* genotypes within the group with 2 *APOL1* RAs were composed of 13 G1G1, 13 G1G2, and 4 G2G2. All participants with 2 *APOL1* RAs received at least 2 doses of vaccine. Most (90%) received the BNT162B2 (Pfizer) or mRNA-1273 (Moderna) vaccine for their initial and subsequent doses. Of the 3 high-risk participants who initially received the Ad26.COV2.S (Johnson & Johnson) vaccine, all received an mRNA vaccine at their subsequent dose. Vaccines were administered between December 2020 and December 2022 for all participants. All laboratory data for the group with 2 APOL1 RAs were collected after the second or later dose of vaccine, with a median timepoint of 117 days from last dose. In the group with 1 *APOL1* RA, median time point from vaccine was 68 and 120 days for serum creatinine and urine albumin-to-creatinine ratio, respectively; and 105 and 94 days for the group with 0 *APOL1* RA. Individual participant demographics, laboratory data, and summary characteristics were similar across groups and are presented in Table 1 and Supplementary Table S1.

**Table 1:** Descriptive characteristics and clinical data of study participants stratified by number of APOL1 risk alleles

Characteristics	2 Risk Alleles	1 Risk Allele	0 Risk Alleles	*P*-value
	(*n* = 30)	(*n* = 39)	(*n* = 36)	
Mean age (range), yr	63 (42–81)	61 (35–85)	63 (32–83)	0.77
Male sex, *n* (%)	7 (23)	11 (28)	11 (31)	0.82
Race, *n* (%)				
African American/Black	30 (100)	39 (100)	36 (100)	>0.99
Vaccine doses ≥ 2, *n* (%)	30 (100)	37 (95)	32 (89)	0.20
mRNA vaccine, *n* (%)	30 (100)	38 (97)	35 (97)	>0.99
Serum creatinine, mg/dl, mean, SD (*n*)				
Baseline	0.92, 0.18 (26)	0.93, 0.24 (39)	0.97, 0.20 (36)	0.63
Postvaccine	0.92, 0.18 (30)	0.92, 0.25 (39)	0.95, 0.17 (36)	0.51
Urine protein, mean, SD (*n*)				
UPCR, mg/g				
Baseline	106, 49 (17)			
Postvaccine	105, 47 (20)			
ACR, mg/g				
Baseline	111, 70 (4)	194, 87 (13)	112, 89 (19)	0.02^a^
Postvaccine	176, 94 (5)	139, 78 (15)	100, 62 (19)	0.09
Timepoint postvaccine (days from vaccine to lab draw)				
Serum creatinine: median days, SD (range)	117, 128 (3–631)	68, 89 (2–420)	105, 121 (2–490)	0.11
Urine albumin/creatinine: median days, SD (range)	117, 132 (3–631)	120, 112 (1–459)	94, 126 (4–433)	0.86

ACR, urine albumin-to-creatinine ratio; UPCR, urine protein-to-creatinine ratio.

a *P* < 0.05.

### *APOL1* HRG Did Not Associate With New Onset Proteinuria or Increase in Serum Creatinine Following SARS-CoV-2 Vaccination in Our Cohort

**Figure 2:**
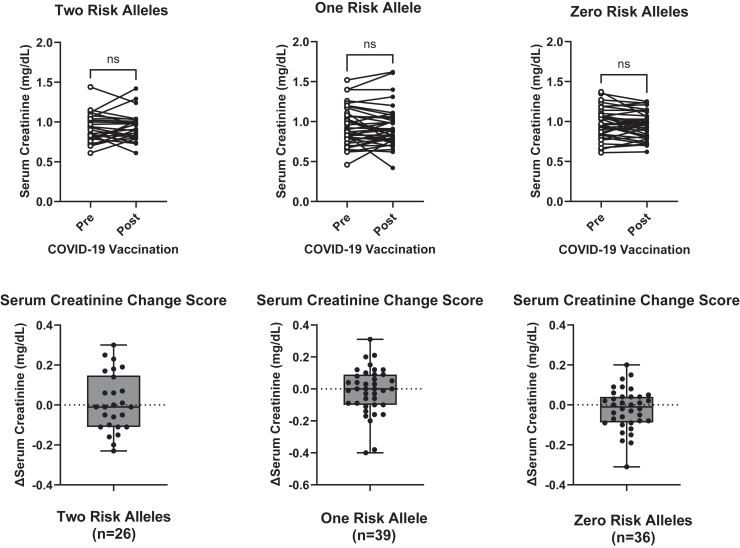
Paired prevaccine and postvaccine serum creatinine measures stratified by number of *APOL1* risk alleles. Comparison of means was performed in each group using the Wilcoxon signed rank test with significance set at *P* < 0.05. Box plot of change score for each participant (prevaccine value subtracted from postvaccine value) illustrating distribution around 0. Four participants in the 2 risk allele group were excluded from analysis due to available baseline data falling after vaccination. Two risk alleles (*n* = 26), 1 risk allele (*n* = 39), 0 risk alleles (*n* = 36). *APOL1*, Apolipoprotein L1; ns, not significant.

**Figure 3:**
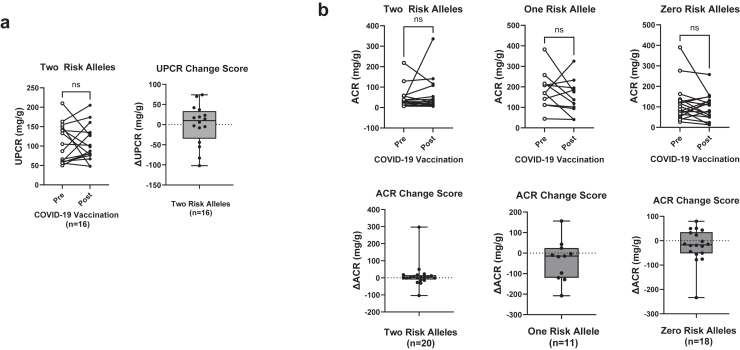
(a) Paired measures of urine protein-to-creatinine ratio (mg/g) obtained prevaccine and postvaccine from high-risk participants in DARB cohort and blox-plot of change score for each participant (prevaccine value subtracted from postvaccine value) illustrating distribution around 0 (*n* = 16). (b) Paired prevaccine and postvaccine urine albumin-to-creatinine ratios (mg/g) stratified by number of *APOL1* risk alleles. For the 16 two risk allele participants with only paired measures of UPCR (graphed in a), albuminuria levels were estimated using proteinuria via the previously published conversion equation by Sumida *et al.*^28^ Box-plot of change score for each participant (prevaccine value subtracted from postvaccine value) illustrating distribution around 0. Comparison of means was performed using the Wilcoxon signed rank test with significance set to *P* < 0.05. Two risk alleles (*n* = 20), 1 risk allele (*n* = 11), 0 risk alleles (*n* = 18). ACR, urine albumin-to-creatinine ratio; DARB, Duke APOL1 Research Biorepository; ns, not significant; UPCR, urine protein-to-creatinine ratio.

**Figure 4:**
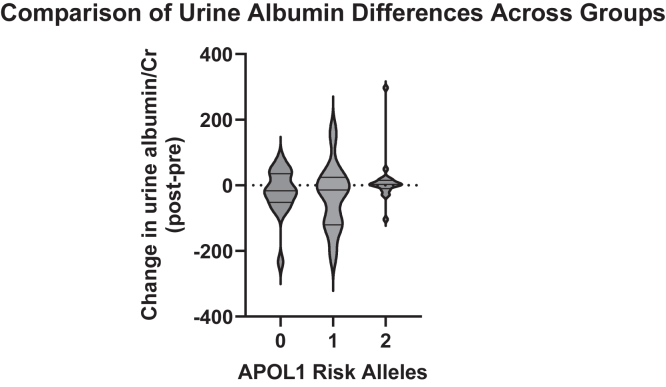
Comparison of urine protein differences across groups graphed as a violin plot showing median, quartiles, minimum, and maximum values. Comparison across strata using 1-way analysis of variance found no significant difference, significance set at *P* < 0.05. Two risk alleles (*n* = 20), 1 risk allele (*n* = 11), 0 risk alleles (*n* = 18).

No significant difference was found between prevaccine and postvaccine serum creatinine in the 2 RA group (*n* = 26), the 1 RA group (*n* = 39), or the 0 RA group (*n* = 36) (Figure 2). Paired prevaccine and postvaccine UPCR (mg/g) measures obtained from high-risk participants were sufficient for comparison in 16 cases. Using a Wilcoxon signed rank test, we found no significant difference in proteinuria among 2 *APOL1* RA individuals following administration of COVID-19 vaccine (Figure 3a). Paired measures of urine albumin-to-creatinine ratio (mg/g) were available for 4 participants with 2 *APOL1* RAs, 11 participants with 1 *APOL1* RA, and 18 participants with 0 *APOL1* RA. For the 16 participants in the group with 2 APOL1 RAs with only paired measures of UPCR, albuminuria levels were estimated using proteinuria measurements via a previously published conversion equation by Sumida *et al.*^28^ for the purpose of additional inclusion in comparative analysis across groups.^29^ Similar to the UPCR in the 2 *APOL1* RA group, there was no difference in urine albumin-to-creatinine ratio within the groups for 1 *APOL1* RA, 0 *APOL1* RA, or in the 2 *APOL1* RA group (Figure 3b). Change scores were additionally calculated for each individual’s paired laboratory measurements (prevaccine measurement subtracted from postvaccine measurement). There were no significant differences in change scores across the 3 groups when analyzed using analysis of variance (Figure 4).

### *APOL1* Haplotype Background Does Not Explain the Lack of New-Onset Glomerular Disease Following SARS-CoV-2 Vaccination

**Figure 5:**
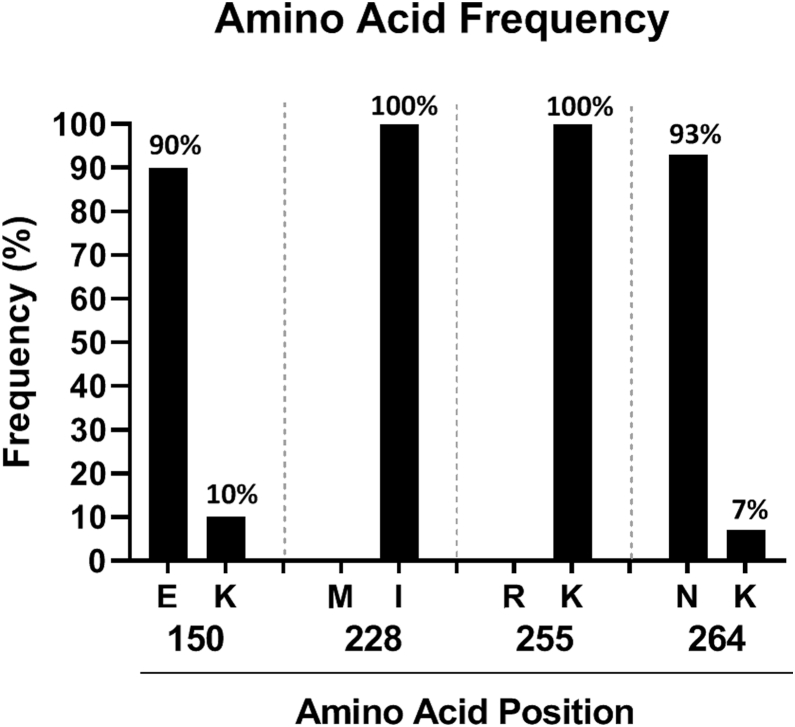
Amino acid frequencies at positions 150, 228, 255, and 264. Most participants have G1 and G2 alleles on the most common haplotype background which is not associated with reduced toxicity.

Several studies have shown that the cytotoxicity of G1 and G2 *APOL1* is impacted by the haplotype backgrounds on which they occur.^30^, ^31^, ^32^ G1 or G2 with K as the amino acid at position 264 have attenuated toxicity compared to G1 or G2 with N. Variant p.N264K has been shown to reduce cytotoxicity *in vitro* in HEK293 cells with either G1 or G2 RAs.^30^ Homozygous p.N264K substitution has also been shown to reduce the trypanolytic function of APOL1 *in vivo*,^32^ and recently, Hung *et al.*^33^ reported that p.N264K attenuates the association of *APOL1* HRG with chronic kidney disease and end-stage renal disease in a cross-sectional analysis of 121,492 participants of African ancestry from the Million Veteran Program and replicated these findings in the Vanderbilt BioVU biobank. Similar protective effects of p.K264 haplotype was discovered against APOL1-mediated focal segmental glomerulosclerosis.^34^ To evaluate if the less toxic G1 or G2 on p.K264 haplotype background explained renal protection in our high risk cohort, we examined the *APOL1* gene sequence from the 21 *APOL1* HRG DARB participants for whom these data were available. We found that 18 of the 21 participants were homozygous for the reference p.N264. Three participants with G1G2 genotype were heterozygous at this amino acid position, with 1 *APOL1* allele on N264 and the other on K264. None of the participants were homozygous for the protective K264. This result suggests that the absence of kidney disease in these participants was not due to the presence of the less toxic *APOL1* haplotype. Moreover, we confirmed that nearly all the participants carry G1 or G2 on the most common haplotype background (E150, I228, and K255) as previously reported.^31^ (Table 2 and Figure 5).

**Table 2:** APOL1 genotypes and haplotype backgrounds for the 21 study participants with available next-generation sequencing data. Single nucleotide polymorphisms (SNPs) are reported by their resultant amino acids in positions E150K, M228I, R255K, and N264K

Study ID	APOL1 Genotype	E150K	I228M	K255R	N264K
4	G1G2	KE	II	KK	NK
11	G1G1	EE	II	KK	NN
12	G1G1	EE	II	KK	NN
14	G1G2	EE	II	KK	NN
21	G1G1	EE	II	KK	NN
32	G1G1	EE	II	KK	NN
36	G1G2	KE	II	KK	NK
42	G2G2	KE	II	KK	NN
51	G1G2	EE	II	KK	NN
59	G1G1	EE	II	KK	NN
75	G1G1	EE	II	KK	NN
78	G1G2	EE	II	KK	NN
88	G1G1	EE	II	KK	NN
90	G1G2	EE	II	KK	NN
92	G2G2	EE	II	KK	NN
97	G1G2	EE	II	KK	NN
102	G1G2	EE	II	KK	NN
128	G1G1	EE	II	KK	NN
153	G1G1	EE	II	KK	NN
158	G1G1	EE	II	KK	NN
169	G1G2	KE	II	KK	NK

## Discussion

**Figure 6:**
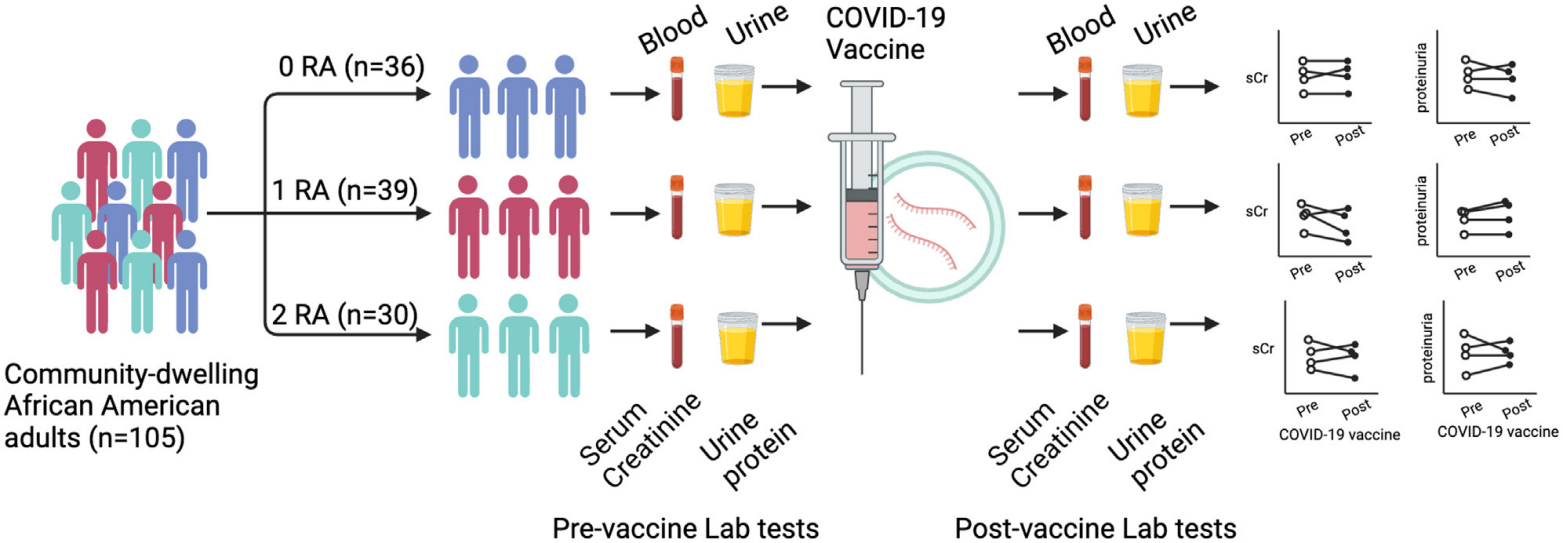
Summary figure and schematic of study. RA, risk allele.

In this study, we report that in a community cohort of African American adults with no significant kidney disease at baseline, *APOL1* HRG was not associated with new onset kidney disease following SARS-CoV-2 vaccination. These conclusions were based on assessment of paired measures of prevaccine and postvaccine serum creatinine and urine protein quantification data within *APOL1* HRG individuals and when compared to participants with 1 or 0 APOL1 RA (Figure 6).

Our study focused on self-identified African American/Black participants who were underrepresented in previous studies that investigated a potential correlation between SARS-CoV-2 vaccine and new or relapsing glomerular disease. For individuals with *APOL1* HRG, conditions that increase cytokines can result in podocytopathy, glomerular disease, and proteinuria. The most clinically relevant second hits found in the literature are parenteral interferon therapy resulting in collapsing glomerulopathy and viral infections, including SARS-CoV-2 infection resulting in COVAN.^16^^,^^17^^,^^20^^,^^21^ Importantly, 2 cases of collapsing glomerulopathy occurring in individuals with *APOL1* HRG following SARS-CoV-2 vaccination have been reported.^6^ Nonetheless, the temporal correlation alone in these 2 cases is not sufficient proof of causality. The knowledge that individuals with *APOL1* HRG have increased risk of developing idiopathic focal segmental glomerulosclerosis, including with collapsing variant, predates the COVID-19 pandemic. In addition, the lack of severe clinical symptoms in these cases does not exclude the possibility that a recent SARS-CoV-2 infection may have triggered the collapsing glomerulopathy. The absence of proteinuria or increase in serum creatinine following SARS-CoV-2 vaccine in 105 community dwelling African Americans in our study, irrespective of their *APOL1* genotype, suggests that SARS-CoV-2 vaccine is unlikely to be a common second hit trigger of APOL1-mediated kidney disease like interferon gamma and viral infections. Although our results do not rule out rare vaccine events, which are best studied in large population studies and through postmarket monitoring such as the Vaccine Adverse Event Reporting System, our results should provide some reassurance to the community regarding vaccine safety for kidney health in African American and Black adults.

The lack of association between *APOL1* HRG and new-onset glomerular disease in the present study follows a recent retrospective cohort study that evaluated risk of any glomerular disease relapse after SARS-CoV-2 vaccination. In this study, Canney *et al.*^4^ found that patients with a history of stable, biopsy-proven glomerulonephritis, including focal segmental glomerulosclerosis, minimal change disease, IgA nephropathy, lupus nephritis, antineutrophilic cytoplasmic antibody-related glomerulonephritis, and membranous nephropathy, had an increased risk of glomerulonephritis relapse after the second or third dose of SARS-CoV-2 vaccine (hazard ratio, 2.23). However, the absolute risk of relapse remained low (1%–5%).^4^ Notably, participants in the study by Canney *et al.*^4^ had preexisting glomerular disease, which may predispose to relapse during immune activation. Case reports of glomerulonephritis in patients who were previously without kidney disease in temporal relation to vaccine have also been reported; however, causality of these cases has not been established. In addition, the underlying pathologies described postvaccine and postinfection have differed. IgA nephropathy and minimal change disease were more commonly reported postvaccine and generally had favorable outcomes, whereas more severe phenotypes such as COVAN have been predominantly described post-SARS-CoV-2 infection.^5^^,^^15^^,^^35^ This fact alone may argue against a common renal pathogenesis. Notably, in the recent series describing 28 cases of *de novo* glomerular disease and 1 case of relapse in a transplant recipient in close temporal relationship with SARS-CoV-2 vaccination, the authors observed no overall increase in the incidence of glomerular disease during their study period when compared with the 2 years before the COVID-19 pandemic.^6^

Our result incites the following question. If COVID-19-induced cytokines trigger COVAN in individuals with *APOL1* HRG, why did SARS-CoV-2 vaccine, which also increase systemic cytokines, not increase the incidence of glomerulopathy in individuals with *APOL1* HRG? One hypothesis for this distinction may be the identity of the cytokines, the amplitude of induction, or the duration of the cytokine increase. Previous studies report that COVID-19 causes a sustained elevation of multiple cytokines, including TNF, IL-6, IL-1β, IL-18, and type1 and type2 interferons.^16^^,^^23^^,^^24^^,^^36^ We demonstrated that many of these cytokines synergize to drive expression of pathogenic APOL1 in podocytes and glomerular endothelial cells.^19^ By comparison, SARS-CoV-2 vaccine transiently increases systemic levels of IL-15, IFN-gamma, IL-6, and CXCL10 after first vaccination and TNF after second vaccination.^22^ The differences in the composition, degree of elevation, and duration of these cytokines may explain the differences in clinical phenotype observed under the 2 conditions.

Our study is not without limitations. Although we recruited from a large cohort, our final sample size was small. This increased the risk for type 2 error and could result in sampling bias or unknown confounders in our study population. The relatively normal baseline serum creatinine and the low to normal urine protein levels indicate that our cohort did not have clinically significant kidney disease at baseline. Therefore, our results may best apply to similar individuals in the general population. Because our cohort did not include individuals with *APOL1* HRG with known preexisting glomerular disease, the generalizability of our results to these individuals is uncertain. Postvaccine laboratory data in our *APOL1* HRG group was on average collected 3 to 4 months after the second or later dose of vaccine. Although this follow-up period is longer than the 1 month follow-up described in previous case reports, the validity of our conclusion beyond 4 months postvaccination is unknown. Future studies of larger cohorts and longer follow-up are necessary to validate our findings and further support the renal safety of SARS-CoV-2 vaccine in individuals with *APOL1* HRG.

In summary, we find that in a community cohort of African American and Black adults who received SARS-CoV-2 vaccine, individuals with *APOL1* HRG did not develop a significant worsening of proteinuria or decline in kidney function. These results should induce confidence and decrease perceived barriers to vaccination in persons with *APOL1* HRG. Major priorities of all successful vaccine campaigns are to remove barriers to vaccination, eliminate racial or ethnic disparities, and address vaccine safety issues. This is especially important for patients with risk factors for severe disease such as those with glomerular disease, reduced kidney function, or genetic risk factors for COVID-19 complications such as COVAN, and the well-established benefit of SARS-CoV-2 vaccination.

## Disclosure

OAO is an advisor to Maze Therapeutics, Podium Bio and received research support from Icagen and Eli Lilly. All the other authors declared no competing interests.

## Supplementary Material

Supplementary File (Excel) **Table S1.** Individual participant demographics, vaccine information, and laboratory data.

## Footnotes

Supplementary File (Excel)
**Table S1.** Individual participant demographics, vaccine information, and laboratory data.
