# Supplementary material for: APOL1 High-Risk Genotype is Not Associated With New or Worsening of Proteinuria or Kidney Function Decline Following COVID-19 Vaccination
Source: Kidney Int Rep. 2024 Jun 20;9(9):2657–66. doi: 10.1016/j.ekir.2024.06.023 (PMC11403097; doi:10.1016/j.ekir.2024.06.023)
Supplement: Supplementary File (Excel) — Table S1. Individual participant demographics, vaccine information, and laboratory data. [file mmc1.pdf]

Table Supplement 1.

Study ID	Age	Gender	Genotype	Total Vaccine Doses Received	Vaccine Manufacturer	Vaccine Dates-1	Vaccine Dates-2	Vaccine Dates-3	Vaccine Dates-4	Serum Cr baseline (mg/dL)	Pre-Cr Date	Serum Cr post-vaccine (mg/dL)	Post-Cr Date	Cr Delta (DAYS)		UPCR baseline (mg/g)	Pre-UPCR date	UPCR post-vaccine (mg/g)	Post-UPCR date	Calculated ACR baseline (mg/g)		Calculated ACR post-vaccine (mg/g)		ACR Delta (DAYS)	
4	50	F	G1G2	4	Moderna	3/5/2021	4/2/2021	12/10/2021	5/4/2022	0.85	11/5/2020	0.74	6/2/2022	29	after 4th vaccine dose	66	11/5/2020	80	6/2/2022	9	11/5/2020	12	6/2/2022	29	after 4th vaccine dose
11	54	F	G1G1	3	Pfizer	4/12/2021	5/4/2021	3/26/2022		0.93	11/13/2020	0.82	6/2/2022	68	after 3rd dose	87	11/13/2020	161	6/2/2022	14	11/13/2020	37	6/2/2022	68	after 3rd dose
12	64	F	G1G1	2	Johnson & Johnson/ Pfizer	5/10/2021	3/1/2022			1	11/20/2020	0.99	5/31/2022	91	after 2nd dose	101	11/20/2020	Insufficient sample	5/31/2022	18	11/20/2020	Insufficient sample	5/31/2022	91	after 2nd dose
14	52	F	G1G2	2	Pfizer	9/1/2021	10/1/2021			0.79	12/14/2020	0.73	6/17/2022	259	after 2nd dose	58	12/14/2020	81	6/17/2022	7	12/14/2020	12	6/17/2022	259	after 2nd dose
21	70	M	G1G1	4	Pfizer	1/23/2021	2/13/2021	9/30/2021	4/23/2022	1.06	11/13/2020	1.29	6/7/2022	45	after 4th vaccine dose	163	11/13/2020	205	6/7/2022	37	11/13/2020	54	6/7/2022	45	after 4th vaccine dose
32	73	F	G1G1	2	Pfizer	8/16/2021	9/7/2021			0.94	12/18/2020	0.79	6/2/2022	268	after 2nd dose	61	12/18/2020	131	6/2/2022	8	12/18/2020	26	6/2/2022	268	after 2nd dose
36	73	M	G1G2	4	Pfizer	1/23/2021	2/13/2021	9/8/2021	4/12/2022	1.02	1/11/2021	1.01	6/9/2022	58	after 4th vaccine dose	133	1/11/2021	78	6/9/2022	27	1/11/2021	12	6/9/2022	58	after 4th vaccine dose
42	74	F	G2G2	4	Moderna	1/21/2021	2/18/2021	11/8/2021	6/27/2022	0.71	9/18/2012	0.9	1/23/2023	210	after 4th vaccine dose	105	9/18/2012	102	1/23/2023	19	9/18/2012	18	1/23/2023	210	after 4th vaccine dose
51	68	F	G1G2	3	Moderna	2/20/2021	3/10/2021	3/1/2022		0.61	2/4/2021	0.79	7/28/2022	149	after 3rd dose	142	2/4/2021	98	7/28/2022	30	2/4/2021	17	7/28/2022	149	after 3rd dose
59	54	M	G1G1	5	Pfizer	12/28/2021	1/19/2022			0.94	2/16/2021	0.89	7/26/2022	188	after 2nd dose	59	2/16/2021	76	7/26/2022	8	2/16/2021	11	7/26/2022	188	after 2nd dose
75	55	F	G1G1	4	Moderna	1/25/2021	2/23/2021	11/3/2021	4/28/2022			0.59	6/7/2022	40	after 4th vaccine dose			75	6/7/2022			11	6/7/2022	40	after 4th vaccine dose
78	54	F	G1G2	2	Pfizer	10/4/2021	10/25/2021			0.8	3/31/2021	0.97	6/17/2022	235	after 2nd dose	140	3/31/2021	135	6/17/2022	29	3/31/2021	28	6/17/2022	235	after 2nd dose
88	73	M	G1G1	4	Pfizer	3/7/2021	3/28/2021	10/19/2021	4/12/2022			0.99	6/14/2022	63	after 4th vaccine dose			190	6/14/2022			48	6/14/2022	63	after 4th vaccine dose
90	61	F	G1G2	2	Pfizer	4/22/2021	5/12/2021			0.73	4/9/2021	0.8	2/2/2023	631	after 2nd dose	210	4/9/2021	127	2/2/2023	56	4/9/2021	25	2/2/2023	631	after 2nd dose
92	57	F	G2G2	2	Johnson & Johnson/ Moderna	3/1/2021	11/15/2021			0.9	7/6/2012	0.8	6/30/2022	227	after 2nd dose	61	7/6/2012	67	6/30/2022	8	7/6/2012	9	6/30/2022	227	after 2nd dose
97	56	F	G1G2	4	Pfizer	3/19/2021	5/1/2021	11/8/2021	6/13/2022	0.8	4/15/2014	0.86	6/21/2022	8	after 4th vaccine dose	150	4/15/2014	48	6/21/2022	33	4/15/2014	6	6/21/2022	8	after 4th vaccine dose
102	57	F	G1G2	2	Johnson & Johnson/ Pfizer	4/5/2021	1/19/2022			0.76	3/7/2015	1.01	6/17/2022	149	after 2nd dose	50	3/7/2015	87	6/17/2022	6	3/7/2015	14	6/17/2022	149	after 2nd dose
128	69	M	G1G1	4	Moderna/ Pfizer	3/4/2021	4/1/2021	12/1/2021	4/23/2022	1.12	3/3/2012	1.42	6/28/2022	66	after 4th vaccine dose	56	3/3/2012	48	6/28/2022	7	3/3/2012	6	6/28/2022	66	after 4th vaccine dose
153	52	F	G1G1	4	Pfizer	3/1/2021	3/21/2021	12/23/2021	6/4/2022			0.9	6/30/2022	26	after 4th vaccine dose			61	6/30/2022			8	6/30/2022	26	after 4th vaccine dose
158	72	F	G1G1	4	Pfizer	2/25/2021	3/18/2021	10/5/2021	4/6/2022			0.86	7/21/2022	106	after 4th vaccine dose			75	7/21/2022			11	7/21/2022	106	after 4th vaccine dose
169	52	F	G1G2	5	Pfizer	3/5/2021	4/1/2021	9/10/2021	6/16/2022	0.7	6/5/2013	0.84	1/25/2023	223	after 4th vaccine dose	155	6/5/2013	174	1/25/2023	35	6/5/2013	41	1/25/2023	223	after 4th vaccine dose
						Vaccine Dates-1	Vaccine Dates-2	Vaccine Dates-3	Vaccine Dates-4		Pre-Cr Date		Post-Cr Date	Cr Delta (DAYS)						ACR baseline (mg/g)	Pre-ACR date	ACR post-vaccine (mg/g)	Post-ACR date	ACR Delta (DAYS)	
500	75	F	G1/G2	3	Pfizer	2/6/2021	2/27/2021	10/4/2021		0.97	7/30/2020	0.98	9/25/2021	210	after 2nd vaccine dose										
501	61	M	G1/G2	3	Pfizer	2/25/2021	3/14/2021	11/10/2021		1.08	12/29/2020	1.03	12/29/2021	49	after 3rd vaccine dose										
502	73	F	G1/G2	4	Pfizer	3/10/2021	3/16/2021	3/31/2021	4/6/2021	1.44	7/14/2020	1.24	8/11/2021	127	after 4th dose					218.7	7/14/2020	115	8/11/2021	127	after 4th vaccine dose
503	75	F	G1/G2	3	Moderna	3/11/2021	4/7/2021	12/1/2021		1.04	1/23/2020	1.04	5/25/2021	48	after 2nd dose					58.5	1/23/2020	108.2	5/25/2021	48	after 2nd dose
504	55	M	G1/G1	2	Pfizer	3/9/2021	3/30/2021			1.12	6/11/2020	0.96	11/16/2021	231	after 2nd dose					39	11/13/2020	336	11/16/2021	231	after 2nd dose
505	43	F	G2/G2	2	Pfizer	3/9/2021	3/30/2021			0.84	3/18/2020	0.61	2/5/2022	312	after 2nd dose										
506	81	F	G2/G2	2	Pfizer	3/5/2021	3/26/2021			0.83	6/4/2020	0.82	5/28/2021	63	after 2nd dose					129	11/15/2020	140.9	11/19/2021	238	after 2nd dose
507	65	F	G1/G1	2	Moderna	3/26/2021	4/23/2021			0.85	2/1/2020	0.91	4/26/2021	3	after 2nd dose										
508	63	F	G1/G1	3	Moderna	5/16/2021	6/13/2021	12/20/2021		1.15	2/7/2020	1.04	7/13/2022	205	after 3rd dose					na		181.3	12/23/2021	3	after 3rd dose
509	66	F	G0/G2	3	Pfizer	3/23/2021	4/13/2021	10/25/2021		1.11	5/27/2020	0.73	7/15/2021	93	after 2nd dose					169.4	5/26/2020	40.7	4/14/2021	1	after 2nd dose
510	70	F	G0/G1	1	Johnson & Johnson	3/13/2021				0.67	2/20/2020	0.79	12/8/2021	270	after solo dose (J&J)					111.4	9/23/2020	94.1	8/11/2021	151	after solo dose (J&J)
511	61	F	G0/G2	2	Pfizer	5/18/2021	6/8/2021			0.7	3/13/2020	0.7	6/25/2021	17	after 2nd dose										
512	41	F	G0/G1	2	Moderna	2/12/2021	3/12/2021			0.65	5/21/2020	0.62	5/6/2022	420	after 2nd dose										
513	44	F	G0/G1	2	Moderna	1/30/2021	2/27/2021			0.62	2/13/2020	0.71	9/14/2021	199	after 2nd dose										
514	36	M	G0/G1	2	Pfizer	4/25/2021	Unknown			1.26	11/7/2020	1.2	7/15/2021	81	after first dose					141.3	11/8/2020	184.3	7/28/2022	459	after first dose
515	85	F	G0/G1	3	Pfizer	2/2/2021	2/25/2021	11/27/2021		1.52	1/25/2020	1.62	4/27/2021	61	after 2nd dose					207.8	5/20/2020	232.6	6/25/2021	120	after 2nd dose
516	75	M	G0/G2	4	Moderna	2/5/2021	3/6/2021	11/18/2021	6/28/2022	1.03	7/9/2020	0.83	7/15/2021	131	after 2nd dose										
517	61	M	G0/G1	3	Moderna	3/29/2021	4/26/2021	11/20/2021		1.23	2/15/2020	1.31	4/30/2021	4	after 2nd dose					382.6	1/30/2021	175	4/2/2022	133	after 3rd dose
518	60	F	G0/G1	3	Pfizer	8/11/2021	9/1/2021	9/28/2021		1.06	3/6/2021	1.06	9/22/2021	21	after 2nd dose										
519	62	F	G0/G1	3	Pfizer	3/13/2021	4/10/2021	1/10/2022		0.9	1/21/2020	0.99	10/6/2021	179	after 2nd dose										
520	54	F	G0/G2	2	Pfizer	3/10/2021	3/31/2021			0.92	2/25/2020	0.91	5/5/2021	35	after 2nd dose										
521	69	M	G0/G2	3	Pfizer	2/12/2021	3/8/2021	12/21/2021		1.4	1/28/2021	1.61	1/29/2022	39	after 3rd dose										
522	70	F	G0/G1	4	Pfizer	1/23/2021	2/13/2021	9/28/2021	5/3/2022	0.79	2/9/2020	0.69	3/17/2021	32	after 2nd dose					168.6	1/5/2021	325.2	10/28/2021	30	after 3rd dose
523	65	F	G0/G2	4	Pfizer	4/2/2021	4/25/2021	10/8/2021	4/13/2022	0.62	2/17/2021	0.65	7/7/2021	73	after 2nd dose					na		39.9	12/9/2021	62	after 3rd dose
524	63	M	G0/G1	2	Pfizer	2/27/2021	3/21/2021			1.19	6/11/2020	1.08	10/1/2021	194	after 2nd dose					293.4		na			
525	35	F	G0/G1	3	Pfizer	2/20/2021	3/13/2021	1/4/2022		0.82	6/25/2020	0.42	5/5/2022	121	after 3rd dose										
526	46	F	G0/G1	2	Pfizer	8/31/2021	9/21/2021			0.81	11/18/2020	0.75	11/24/2021	64	after 2nd dose										
527	63	F	G0/G1	3	Moderna	2/13/2021	3/13/2021	11/9/2021		0.73	1/20/2021	0.78	6/15/2022	218	after 3rd dose					257.7	7/17/2020	137.4	8/5/2021	145	after 2nd dose
528	83	M	G0/G1	2	Pfizer	2/11/2021	3/8/2021			0.74	4/9/2020	0.89	4/22/2021	45	after 2nd dose										
529	72	M	G0/G1	4	Pfizer	3/4/2021	3/26/2021	10/29/2021	6/29/2022	1.07	5/15/2020	1.04	6/9/2021	75	after 2nd dose					209.4	5/15/2020	194.9	6/9/2021	75	after 2nd dose
530	54	F	G0/G1	3	Moderna	3/30/2021	4/27/2021	2/24/2022		0.84	10/3/2020	0.9	2/26/2022	2	after 3rd dose										
531	62	M	G0/G1	4	Pfizer	6/23/2021	7/15/2021	12/8/2021	5/20/2022	0.68	5/20/2020	0.8	9/17/2021	64	after 2nd dose					44.4	2/12/2021	41.2	5/12/2022	155	after 3rd dose
532	75	F	G0/G2	2	Pfizer	6/21/2021	7/20/2021			0.89	4/7/2020	0.73	10/28/2021	100	after 2nd dose										
533	60	F	G0/G1	2	Moderna	3/12/2021	4/5/2021			1.08	1/30/2020	0.98	9/3/2021	151	after 2nd dose					112.1	1/30/2021	101.9	9/3/2021	151	after 2nd dose
534	72	F	G0/G1	2	Pfizer	1/21/2021	2/11/2021			1	2/11/2020	0.83	2/2/2021	12	after first dose					215.9	7/28/2020	118.9	4/20/2021	68	after 2nd dose
535	55	M	G0/G2	2	Johnson & Johnson/ Pfizer	6/8/2021	6/13/2022			0.8	8/7/2020	1	7/11/2022	28	after 2nd dose										
536	40	F	G0/G1	4	Pfizer/Moderna	3/24/2021	4/14/2021	11/17/2021	10/13/2022	1.01	11/20/2020	0.87	12/20/2021	33	after 3rd dose										
537	49	F	G0/G1	3	Pfizer	4/1/2021	4/22/2021	12/1/2021		0.79	1/16/2020	0.78	5/24/2021	32	after 2nd dose										
538	68	F	G0/G1	3	Pfizer	2/17/2021	3/10/2021	11/25/2021		0.86	3/7/2020	0.77	4/30/2021	51	after 2nd dose					na		109.2	4/22/2021	43	after 2nd dose
539	72	F	G0/G1	2	Pfizer	3/5/2021	3/26/2021			0.87	6/3/2020	0.91	12/8/2021	257	after 2nd dose					210.4		na			
540	48	F	G0/G2	3	Pfizer	3/26/2021	4/18/2021	11/2/2021		0.46	7/20/2020	0.77	8/31/2021	135	after 2nd dose										
20	67	F	G1/G0	4	Pfizer	4/13/2021	5/4/2021	11/11/2021	7/19/2022	0.96	2/13/2020	0.99	5/12/2021	8	after 2nd dose					na		169.2	6/30/2022	231	after 3rd dose
45	60	F	G1/G0	2	Moderna	2/28/2021	3/28/2021			1.17	7/15/2020	1.01	6/4/2021	68	after 2nd dose										
24	57	F	G1/G0	3	Moderna	2/19/2021	3/18/2021	11/16/2021		0.83	6/26/2020	0.83	3/19/2022	123	after 3rd dose										
174	71	F	G0/G2	3	Pfizer	2/19/2021	3/12/2021	Unknown		1.4	3/11/2020	1.4	4/14/2021	33	after 2nd dose					na		115.4	4/14/2021	33	after 2nd dose
80	72	M	G1/G0	1	Pfizer	2/5/2021				1.2	5/20/2020	1.11	2/28/2021	23	after first dose										
2	58	M	G1/G0	3	Pfizer/Moderna	3/3/2021	3/24/2021	12/6/2021		0.98	2/19/2020	1.1	6/4/2021	72	after 2nd dose										
39	71	F	G1/G0	4	Moderna	2/17/2021	3/17/2021	10/27/2021	12/16/2022	0.78	12/16/2020	0.82	3/19/2022	143	after 3rd dose										
541	53	M	G0/G0	2	Pfizer	4/28/2021	5/2/2021			1.03	9/15/2020	0.84	10/13/2021	164	after 2nd dose					51.8	9/14/2020	131.5	3/16/2022	318	after 2nd dose
542	50	F	G0/G0	2	Pfizer	3/20/2021	4/20/2021			0.84	2/25/2020	0.79	5/20/2021	30	after 2nd dose					162.7	8/19/2020	117.9	5/19/2021	29	after 2nd dose
543	70	F	G0/G0	1	Pfizer	9/13/2021				0.96	1/6/2020	0.97	11/20/2021	68	after first dose					137.4	3/31/2021	59.1	8/17/2022	338	after first dose
544	56	M	G0/G0	3	Moderna	12/31/2020	1/28/2021	11/3/2021		1.03	11/17/2020	1.03	3/17/2021	48	after 2nd dose										
545	53	F	G0/G0	2	Moderna	3/18/2021	4/15/2021			1.06	1/29/2020	1	3/23/2022	342	after 2nd dose					63.9	1/29/2020	50	6/22/2022	433	after 2nd dose
546	47	F	G0/G0	1	Johnson & Johnson	8/17/2021				0.92	1/29/2020	0.96	2/25/2022	192	after first dose										
547	62	M	G0/G0	2	Johnson & Johnson/ Moderna	2/27/2021	11/24/2021			0.84	6/20/2020	1.04	6/4/2022	192	after 2nd dose					118.3	7/18/2020	150.8	6/4/2022	192	after 2nd dose
548	50	F	G0/G0	3	Pfizer	3/18/2021	4/8/2021	12/21/2021		0.65	8/28/2020	0.78	8/30/2021	144	after 2nd dose										
549	62	F	G0/G0	3	Pfizer	3/25/2021	4/23/2021	12/29/2021		0.83	7/29/2020	0.74	12/3/2021	224	after 2nd dose										
550	53	F	G0/G0	3	Moderna	4/2/2021	4/30/2021	12/10/2021		0.8	2/8/2020	0.72	4/2/2022	113	after 3rd dose										
551	83	F	G0/G0	3	Moderna	2/14/2021	3/13/2021	11/20/2021		0.67	3/10/2020	0.72	3/18/2021	5	after 2nd dose					43.5	3/9/2020	24	3/17/2021	4	after 2nd dose
552	62	M	G0/G0	2	Moderna	5/21/2021	6/19/2021			1.19	2/21/2020	1.12	9/24/2021	97	after 2nd dose					389.6	1/12/2021	156.2	9/21/2021	94	after 2nd dose
553	77	M	G0/G0	2	Moderna	1/27/2021	2/24/2021			1.14	6/3/2020	1.14	6/18/2021	114	after 2nd dose										
554	65	F	G0/G0	2	Moderna	3/17/2021	4/14/2021			0.72	1/31/2020	0.7	8/17/2022	490	after 2nd dose					79.1	5/15/2020	128.8	4/14/2021	28	after first dose
555	74	M	G0/G0	2	Pfizer	2/13/2021	3/6/2021			1.24	2/9/2021	1.15	6/10/2021	96	after 2nd dose					27	2/9/2021	12.5	6/10/2021	96	after 2nd dose
556	60	F	G0/G0	3	Pfizer	1/21/2021	2/12/2021	7/26/2022		0.61	10/22/2020	0.62	3/31/2022	412	after 2nd dose										
557	58	M	G0/G0	2	Moderna	2/17/2021	3/17/2021			1.1	12/2/2020	1.07	6/5/2021	80	after 2nd dose					101.9	2/20/2020	51.2	6/8/2021	83	after 2nd dose
558	72	M	G0/G0	3	Pfizer	2/13/2021	3/6/2021	11/12/2021		1.19	12/29/2020	1.21	4/30/2021	55	after 2nd dose					66.8	12/29/2020	117.5	4/30/2021	55	after 2nd dose
559	69	F	G0/G0	3	Moderna	2/14/2021	3/14/2021	12/12/2021		0.95	10/22/2020	0.8	9/16/2021	186	after 2nd dose					na		160.1	4/1/2021	18	after 2nd dose
560	63	F	G0/G0	2	Moderna	3/25/2021	4/22/2021			0.77	12/5/2020	0.81	12/9/2021	231	after 2nd dose					27.7		na			
561	64	F	G0/G0	4	Moderna/ Pfizer	4/7/2021	5/5/2021	12/20/2021	7/15/2022	0.96	5/20/2020	0.82	7/30/2021	86	after 2nd dose										
562	70	F	G0/G0	3	Moderna	1/30/2021	2/27/2021	1/5/2022		0.67	2/20/2020	0.71	4/2/2021	34	after 2nd dose										
563	65	M	G0/G0	2	Pfizer	3/24/2021	4/14/2021			1.27	9/5/2020	1.23	9/10/2021	149	after 2nd dose										
564	79	F	G0/G0	2	Pfizer	2/13/2021	3/6/2021			1.06	3/18/2020	0.88	8/16/2021	163	after 2nd dose					116.3	3/17/2020	61	8/19/2021	166	after 2nd dose
565	76	F	G0/G0	3	Pfizer	1/23/2021	2/13/2021	10/20/2021		1.04	3/5/2020	0.96	3/16/2021	31	after 2nd dose					126	7/16/2020	149.4	3/16/2021	31	after 2nd dose
566	62	F	G0/G0	2	Pfizer	4/1/2021	4/22/2021			1.35	10/17/2020	1.25	11/4/2021	196	after 2nd dose					77.9	10/20/2020	75	11/4/2021	196	after 2nd dose
567	51	F	G0/G0	3	Moderna	4/30/2021	5/28/2021	6/13/2022		0.97	4/30/2020	0.99	6/3/2022	371	after 2nd dose										
568	75	F	G0/G0	2	Pfizer	2/27/2021	3/27/2021			1.08	1/23/2020	0.96	6/17/2021	82	after 2nd dose										
569	71	F	G0/G0	3	Pfizer	3/8/2021	3/30/2021	10/6/2021		0.91	3/11/2020	0.89	5/18/2021	49	after 2nd dose					130.7	10/27/2020	108.2	10/28/2021	22	after 3rd dose
570	61	F	G0/G0	3	Moderna	2/3/2021	3/3/2021	11/4/2021		0.77	8/28/2020	0.92	9/9/2021	190	after 2nd dose					37.9	8/28/2020	81.5	9/9/2021	190	after 2nd dose
571	32	M	G0/G0	1	Moderna	7/26/2021				1.06	6/3/2020	1.15	8/20/2021	25	after first dose					276.5	6/3/2020	257.5	3/31/2022	248	after first dose
27	81	F	G0/G0	2	Moderna	2/5/2021	3/5/2021			0.9	10/31/2020	0.93	2/10/2021	5	after first dose										
15	73	F	G0/G0	2	Pfizer	3/3/2021	3/24/2021			1.37	3/14/2020	1.06	5/11/2021	48	after 2nd dose					88	10/21/2020	12.5	5/11/2021	48	after 2nd dose
83	59	F	G0/G0	3	Pfizer	4/6/2021	4/29/2021	10/22/2021		1.18	1/24/2020	1.24	5/1/2021	2	after 2nd dose										
152	52	F	G0/G0	1	Pfizer	1/6/2021				0.95	1/30/2020	1.03	10/25/2021	292	after first dose										
37	61	M	G0/G0	3	Moderna	3/13/2021	4/10/2021	4/21/2022		0.87	12/18/2020	0.96	5/25/2022	34	after 3rd dose										
